# Serological Evidence for Japanese Encephalitis and West Nile Virus Infections in Domestic Birds in Cambodia

**DOI:** 10.3389/fvets.2020.00015

**Published:** 2020-01-29

**Authors:** Heidi Auerswald, Anne-Sophie Ruget, Helena Ladreyt, Saraden In, Sokthearom Mao, San Sorn, Sothyra Tum, Veasna Duong, Philippe Dussart, Julien Cappelle, Véronique Chevalier

**Affiliations:** ^1^Virology Unit, Institut Pasteur du Cambodge, Institut Pasteur International Network, Phnom Penh, Cambodia; ^2^Epidemiology and Public Health Unit, Institut Pasteur du Cambodge, Institut Pasteur International Network, Phnom Penh, Cambodia; ^3^Centre de Coopération Internationale en Recherche Agronomique pour le Développement (CIRAD), Unité Mixte de Recherche ASTRE, Montpellier, France; ^4^ASTRE, Université Montpellier, CIRAD, INRAE, Montpellier, France; ^5^Epidemiology Unit, Laboratory for Animal Health, French Agency for Food, Environmental and Occupational Health and Safety (ANSES), University Paris-Est, Maisons-Alfort, France; ^6^General Directorate for Animal Health and Production, Ministry of Agriculture, Forestry and Fisheries, Phnom Penh, Cambodia; ^7^National Animal Health and Production Research Institute, General Directorate for Animal Health and Production, Ministry of Agriculture, Forestry and Fisheries, Phnom Penh, Cambodia; ^8^UMR EpiA, INRAE, VetAgro Sup, Marcy lÉtoile, France

**Keywords:** Japanese encephalitis virus, West Nile virus, domestic birds, poultry, Cambodia, serology

## Abstract

Mosquito-borne flaviviruses with an enzootic transmission cycle like Japanese encephalitis virus (JEV) and West Nile virus (WNV) are a major public health concern. The circulation of JEV in Southeast Asia is well-documented, and the important role of pigs as amplification hosts for the virus is long known. The influence of other domestic animals especially poultry that lives in high abundance and close proximity to humans is not intensively analyzed. Another understudied field in Asia is the presence of the closely related WNV. Such analyses are difficult to perform due to the intense antigenic cross-reactivity between these viruses and the lack of suitable standardized serological assays. The main objective of this study was to assess the prevalence of JEV and WNV flaviviruses in domestic birds, detailed in chickens and ducks, in three different Cambodian provinces. We determined the flavivirus seroprevalence using an hemagglutination inhibition assay (HIA). Additionally, we investigated in positive samples the presence of JEV and WNV neutralizing antibodies (nAb) using foci reduction neutralization test (FRNT). We found 29% (180/620) of the investigated birds positive for flavivirus antibodies with an age-depended increase of the seroprevalence (OR = 1.04) and a higher prevalence in ducks compared to chicken (OR = 3.01). Within the flavivirus-positive birds, we found 43% (28/65) with nAb against JEV. We also observed the expected cross-reactivity between JEV and WNV, by identifying 18.5% double-positive birds that had higher titers of nAb than single-positive birds. Additionally, seven domestic birds (10.7%) showed only nAb against WNV and no nAb against JEV. Our study provides evidence for an intense JEV circulation in domestic birds in Cambodia, and the first serological evidence for WNV presence in Southeast Asia since decades. These findings mark the need for a re-definition of areas at risk for JEV and WNV transmission, and the need for further and intensified surveillance of mosquito-transmitted diseases in domestic animals.

## Introduction

Japanese encephalitis virus (JEV) and West Nile virus (WNV) are the most common encephalitic flaviviruses. The family of *Flaviviridae* contains more than 70 members that were originally distinguished based on the cross-reactivity of the antibodies they induce. Early investigations with polyclonal antisera revealed the antigenic relationships and allowed the separation of the mosquito-borne flaviviruses into seven subgroups, called serocomplexes (1, 2). Members of the same serocomplex are defined by the cross-neutralization of the antibodies they induces. JEV and WNV belong to the JEV serocomplex together with other viruses like Murray Valley encephalitis virus (MVEV), St Louis encephalitis virus (SLEV), and Usutu virus (USUV).

Both JEV and WNV share some ecological similarities as they maintain an enzootic transmission cycle with several bird families as natural reservoirs and mosquitoes of the *Culex* species as main vectors (3, 4). Humans and horses are generally considered dead-end hosts, as they do not develop a viremia high enough to infect mosquitoes. An exception are pigs, as they serve as amplification hosts for JEV because they develop sufficient viral titers to support further infection of mosquitoes (5–7). Although the role of ardeid birds as reservoir hosts for JEV is admitted (8, 9), the role of domestic birds as potential amplifying hosts has been little investigated so far. Several surveys implemented in different continents suggest the involvement of domestic birds, especially ducks, in WNV epidemiological cycle, either as an amplifying host or as a reservoir (10–13). With regards to JEV, two experimental studies suggest that young ducks and chickens might produce a sufficient viremia to infect mosquitoes when biting (14, 15). Because of their close association to humans, and the varying levels of seroprevalence observed in domestic birds, their role in the epidemiological cycle as secondary reservoirs may be of importance (16–18).

JEV is mainly found across Eastern, Southern, and Southeastern Asia where it is the most commonly identified pathogen for encephalitis cases in humans (19). Despite the availability of several vaccines since the 1990s, Japanese encephalitis (JE) is still a clinically important disease with around 70,000 cases per year, causing 10,000–15,000 deaths (20–22) and leaving ~30-50% of the survivors with definitive neurological or psychiatric sequelae (4). WNV is nearly globally distributed even if human outbreaks are sporadically reported because fewer than 1% of human WNV infections develop into severe disease (1, 23). However, the impact of WNV on human and animal health increased dramatically during the last two decades, particularly in the United States of America, with more than 2,000 deaths between 1999 and 2018 (24), and in Europe (25, 26). Human WNV cases were also reported in several Asian countries (27–30) but little is known about its epidemiology and its potential impact on health in this continent.

JEV is endemic in Cambodia and the major cause of central nervous system infections leading to encephalitis and other severe clinical outcomes in children (31). In 2007, the estimated clinically-declared JE incidence in the country was 11.1 cases per 100 000 children under 15 years of age (32). A better knowledge of JE epidemiology and areas at risk would help focusing preventive measures, such as vaccination, in the future. Regarding WNV in Cambodia, there is little known besides sporadic findings in the 1960s of WNV and its subtype Kunjin virus (33).

As part of a large research program on JE epidemiology in Cambodia (ComAcross project http://www.onehealthsea.org/comacross), this study aimed to investigate the exposure to JEV and WNV of domestic birds sampled in three different rural provinces in Cambodia. The collected serum samples were analyzed for flavivirus antibodies by hemagglutination inhibition assay (HIA) and subsequently JEV and WNV specific antibodies by foci reduction neutralization test (FRNT).

## Materials and Methods

### Study Design

**Figure 1:**
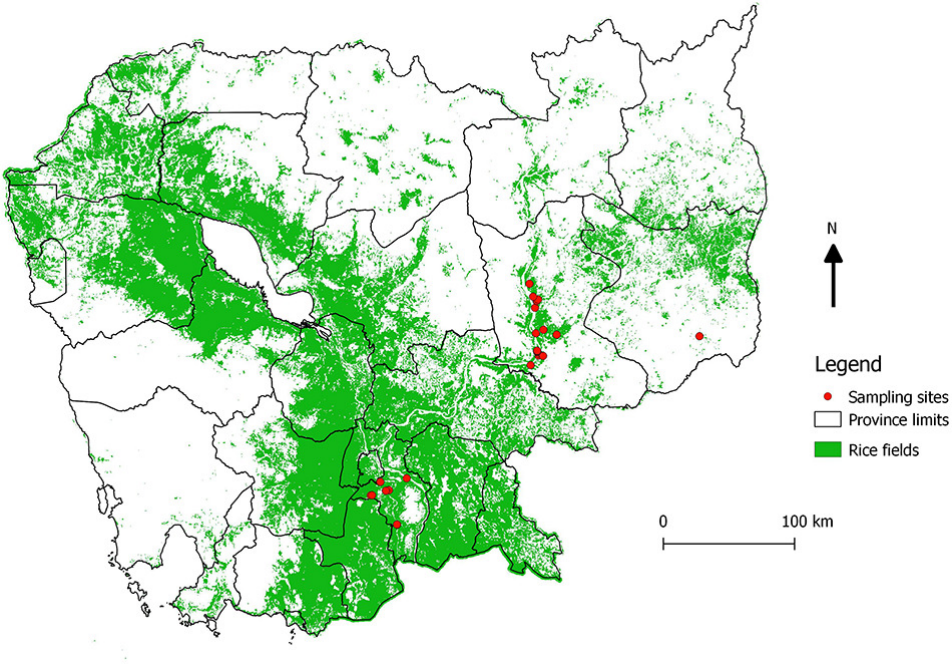
Location of sampling sites. Map showing the locations of the sampled farms (red dots), and the abundance of rice fields (green). The map was created using QGIS 2.14.3 and the base layer data were obtained from DIVA GIS (https://www.diva-gis.org/gdata).

Three geographical areas of Cambodia were selected to get three ecologically contrasted areas according to their landscape characteristics (abundance of rice fields), in addition with their accessibility (distance to Institut Pasteur's laboratory in Phnom Penh) and the ability to provide sufficient number of samples. The fieldwork was conducted in three different provinces in March 2016: Kandal, being a rural area dominated by rice fields, Mondulkiri, mainly dominated by forests and Kratie as an intermediate landscape (Figure 1). The objective was to collect samples from at least ten farms per area to get as much representativity as possible. In each farm, animals were randomly selected and according to the owner agreement.

### Ethics Statement

During this study, we followed the World Animal Health Organization (OIE) guiding principles on animal welfare included in the OIE Terrestrial Animal Health Code (34). All sampling campaigns were implemented with the supervision of the National Animal Health and Production Research Institute (NAHPRI), and local veterinary services.

### Sample Collection

Only chickens and ducks born in the sampling area were included in the study. Blood samples were taken from the ulnar or metatarsal vein. The blood was transferred into serum tube, stored on ice and at 4°C, and centrifuged later (within the sampling day or up to 5 days after sampling depending on the province) to acquire the respective serum sample. Characteristics of the farm and the GPS coordinates of each farm were collected. If known, the age of the birds was given by the farmers.

### Cells and Viruses

Vero cells (ATCC CCL-81) were used for the detection of neutralizing antibodies via FRNT. They were cultivated in Dulbecco's modified Eagle medium (DMEM; Sigma-Aldrich, Steinheim, Germany) supplemented with 10% fetal bovine serum (FBS; Gibco, Gaithersburg, MD, USA) and 100 U/ml penicillin-streptomycin (Gibco) at 37°C and 5% CO_2_ atmosphere. All viruses were grown in C6/36 *Aedes albopictus* cells and harvested from the supernatant. The mosquito cells were cultured in Leibovitz-15 medium (Sigma-Aldrich) supplemented with 10% FBS, 1% L-glutamine (Gibco), 10% tryptose-phosphate (Gibco) and 100 U/ml penicillin-streptomycin at 28°C.

The HIA was performed with the following flavivirus strains: JEV Nakayama (Genbank EF571853), Dengue 2 (DENV-2) strain New Guinea C (Genbank AF038403), Dengue 3 (DENV-3) H87 (Genbank M93130), and Zika (ZIKV) HD78788 (Genbank KF383039, KF383084, KF383047). The FRNT was performed with the above-mentioned JEV reference strain Nakayama and the WNV lineage 1 isolate EG101 (Genbank AF260968).

### Hemagglutination Inhibition Assay (HIA)

The presence of antibodies in the serum samples was analyzed with the HIA using antigen originated from the above-mentioned JEV, DENV-2, DENV-3, and ZIKV strains. The assay followed the protocol previously described (33) adapted to 96 well microtiter plate.

### Foci Reduction Neutralization Test (FRNT)

Due to the high cross-reactivity of the HIA, we aimed to characterize the flavivirus antibodies with a more virus-specific assay. Therefore, we analyzed a subset of 65 sera (39 chicken, 26 duck samples) by foci reduction neutralization tests (FRNTs) against JEV and WNV. The respective samples were chosen because (i) there was sufficient sera volume remaining to perform the FRNT, and (ii) the sample was formerly positive in the HIA for at least one of the tested viruses (JEV, DENV-2, DENV-3, and ZIKV).

The FRNT micro-neutralization assay using reference viruses for JEV and WNV determined the level of neutralizing antibodies and was performed as described previously (35). Briefly, heat inactivated serum samples were analyzed using Vero cells (ATCC CCL-81) seeded in 96 well plates. Serum samples were serial diluted and mixed with equal volume of virus. Virus-serum mixtures were incubated for 1 h at 37°C, and then used for inoculation of Vero cell monolayers. After 1 h of incubation at 37°C on Vero cells, the virus-serum mixtures were replaced by a semi-solid overlay containing 1.6% carboxymethyl cellulose (Sigma-Aldrich) in DMEM medium supplemented with 3% FBS. The plates were incubated at 37°C and 5% CO_2_ atmosphere, and stained the following day. Cells were fixed with 4% paraformaldehyde (Sigma-Aldrich) in phosphate buffered saline (PBS) for 30 min. Afterwards, the plates were incubated sequentially with 0.5% Triton X-100 (Sigma-Aldrich) in PBS for 20 min and with 10% FBS in PBS, polyclonal anti-JEV or anti-WNV mouse hyperimmune ascites fluids (IPC, Cambodia) and anti-mouse IgG antibody conjugated to horseradish peroxidase (Bio-Rad, Marnes La Coquette, France) for 1 h each. Finally, the infected cells were visualized with TrueBlue peroxidase substrate (KPL, Gaithersburg, MD, USA). The amount of neutralizing antibodies (nAb) is expressed as the reciprocal serum dilution that induces 50% reduction of infection visualized as foci (FRNT_50_) compared to the controls (flavivirus-negative control serum and virus dilution without added serum) and was calculated via log probit regression analysis (SPSS for Windows, Version 16.0, SPSS Inc., Chicago, IL, USA). FRNT_50_ titers below 10 were considered negative.

### Statistical Analysis

All FRNT titer calculations were performed as log probit regression by using SPSS for Windows, version 23.0. The statistical analyses were performed using R 3.6.0 (36). The arithmetic means of antibody titers were used for comparative analysis. Given the different diagnostic tests used, we considered the results from the HIA test for flavivirus prevalence, which has been carried out on all samples. Association between seroprevalence and species, age and province was first tested on the whole dataset (*n* = 620) using a Chi-square test. Age was categorized in 3-month increments (Table 1): 1–3 months old, 4–6 months old, 7–9 months old, and 10 months or older. The age was not precisely known for 128 domestic birds, however 22 of these birds which were adults, were categorized as 10 months or older.

**Table 1:** Flavivirus seroprevalence based on hemagglutination inhibition assay (*n* = 620).

	**Samples tested *n* (%)**	HIA positive^*^n (%)			
Total	620	180 (29.0)			
**Province**					
Kandal	296 (47.7)	58 (19.6)			
Kratie	283 (45.6)	98 (34.6)			
Mondulkiri	41 (6.6)	24 (58.5)			
**Species**					
Chicken	417 (67.3)	99 (23.7)			
Duck	203 (32.7)	81 (39.9)			
**Age mean** **(95% CI)**	7.93 (7.19–8.67)		**Age groups**	**Samples tested** ***n*** **(%)**	HIA positive^*^ ***n*** **(%)**
1 month	11 (1.8)	2 (18.2)	1–3 month	195 (31.5)	33 (16.9)
2 months	105 (16.9)	20 (19.0)			
3 months	79 (12.7)	11 (13.9)			
4 months	96 (15.5)	23 (24.0)	4–6 months	144 (23.2)	29 (20.1)
5 months	18 (2.9)	2 (11.1)			
6 months	30 (4.8)	4 (13.3)			
7 months	10 (1.6)	2 (20.0)	7–9 months	28 (4.5)	6 (21.4)
8 months	17 (2.7)	4 (23.5)			
9 months	1 (0.2)	0 -			
10 months	8 (1.3)	4 (50.0)	≥10 months^#^	147 (23.7)	75 (51.0)
11 months	1 (0.2)	0 -			
12 months	32 (5.2)	18 (56.3)			
18 months	9 (1.5)	4 (44.4)			
24 months	57 (9.2)	35 (61.4)			
27 months	2 (0.3)	0-			
30 months	10 (1.6)	0 -			
36 months	6 (1.0)	2 (33.3)			
Unknown ^#^	128 (20.6)	49 (38.3)	Unknown	106 (17.1)	37 (34.9)

* *HIA titer ≥ 80 in at least one of the tested flaviviruses (JEV, DENV-2, DENV-3, ZIKV)*.

# *Including 22 birds with an estimated age of <12 months*.

*HIA Hemagglutination inhibition assay; JEV Japanese encephalitis virus; WNV West Nile virus, DENV dengue virus, ZIKV Zika virus*.

A generalized linear model (glm) was used to assess the link between seroprevalence and age, species and province. Animals for which the exact age was unknown, including all sampled in Mondulkiri, were excluded from the multivariate analysis, and age was used as a discrete variable (age in months). Due to the sampling frame and potential overdispersion, the province (Kandal and Kratie), was incorporated in the model, either as a fixed or a random effect (glmm). The best model was selected according to the Akaike Information Criteria (AIC).

## Results

### Sample Collection

In total, 620 samples were collected (Table 1) in 41 backyard farms with an average of 15 samples per farm. The collection contained 417 (67.3%) blood samples collected from chickens (*Gallus gallus domesticus*) and 203 (32.7%) from ducks (*Anas platyrhynchos domesticus*). In detail, 296 samples (47.7%) were collected in the Kandal province, 283 samples in Kratie (45.6%) and 41 samples (6.6%) in Mondulkiri. The age of the 492 (79.4%) domestic birds could be obtained, and ranged from one to 36 months (mean 7.93 months; 95% CI 7.19–8.67; Supplementary Table 1).

### Flavivirus Seroprevalence Based on HIA

Overall, 180 samples (29%) were detected positive by HIA for at least one of the flaviviruses tested (JEV, DENV-2, DENV-3, and ZIKV) (Table 1). The univariate analysis (Table 2) revealed a significant higher proportion of ducks (39.9%, 81/203 samples) with anti-flavivirus antibodies compared to the amount of positive tested chickens (23.7%; 99/417). The observed flavivirus seroprevalence was also different for the investigated provinces with the lowest seroprevalence in Kandal (19.6%, 58/296 samples), and the highest for Mondulkiri (58.5%; 24/41 samples). Additionally, the seroprevalence rate increased with the age of the birds as antibodies were found in 16.9% (33/195 samples) of young birds (1–3 months old) rising to 51.0% (75/147 samples) in birds that were 10 months or older. For 22.3% (138/620) of the samples, JEV hemagglutinating antibodies were detected (Supplementary Table 1). Also, 157 samples (25.3%) were tested positive for antibodies against DENV-2 and/or DENV-3, and 63 samples (10.2%) against ZIKV. Most of the HIA positive samples showed a positive reaction against more than one of the tested viruses (**Table 4**), as 76.6% (138/180) of the positive samples had antibodies against JEV, and 87.2% (157/180) against DENV-2 and/or DENV-3.

**Table 2:** Results of the univariate analysis (χ2) between seroprevalence and other factors (*n* = 620).

**Variable**	**Df**	***P*-value**
Species	1	3.20e-5
Province	2	3.35e-8
Age group	3	4.79e-12

Regarding the HIA titers for the individual domestic birds, the mean HIA titers differ significantly between the investigated viruses (Supplementary Figure 1). The mean JEV HIA titer was 83.47 (95% CI 58.5-108.5) and therefore significantly lower than for DENV-3 (mean 263.4; 95% CI 146.1–380.8; *p* = 0.0006; Friedman test with Dunn's multiple comparison test). The DENV-3 HIA titers were also significantly higher than for DENV-2 (mean 177.2; 95% CI 111.1-243.4; *p* < 0.0001). Overall, the mean ZIKV HIA was significantly lower than for the other three viruses (mean 39.97; 95% CI 25.28–54.65; *p* < 0.0001). The JEV HIA titers correlated moderate with the HIA titers against the other flaviviruses (DENV-2 *r* = 0.62; DENV-3 *r* = 0.59; ZIKV *r* = 0.65; Supplementary Figure 2). The DENV-2 and DENV-3 titers correlated strongly (*r* = 0.90) but less so with the closely related ZIKV (DENV-2 *r* = 0.53; DENV-3 *r* = 0.49).

According to AIC (AICglm = 553 vs. AICglmm = 558), the best generalized linear model with the flavivirus serological status based on HIA as outcome, and age, species and province as explanatory variables, incorporated the province as a fixed effect. This model confirmed the results of the univariate analysis: the seroprevalence rate is significantly higher in ducks compared to chickens (OR = 3.01, 95%CI: 1.97–4.63) and slightly increased with age (OR = 1.04, 95% CI: 1.0–21.07; Table 3, Figure 2). Domestic birds were also more exposed in Kratie than in Kandal (OR = 2.01, 95%CI: 1.31–3.09).

**Table 3:** Results of generalized linear model (*n* = 492^*^).

**Explanatory variable**	***P*-value**	**OR [IC 95%]**
Intercept	<2e-16	0.11 [0.07–0.17]
Species (ref=chicken)	3.89e-7	3.01 [1.97–4.63]
Province (ref=Kandal)	1.39e-3	2.01 [1.31–3.09]
Age	7.29e-5	1.05^#^ [1.02–1.07]

* *Birds with unknown or only estimated age (e.g., “>12 months”) were removed from the analysis*.

# *Odds ratio of being seropositive for an additional month of age is 1.04*.

**Figure 2:**
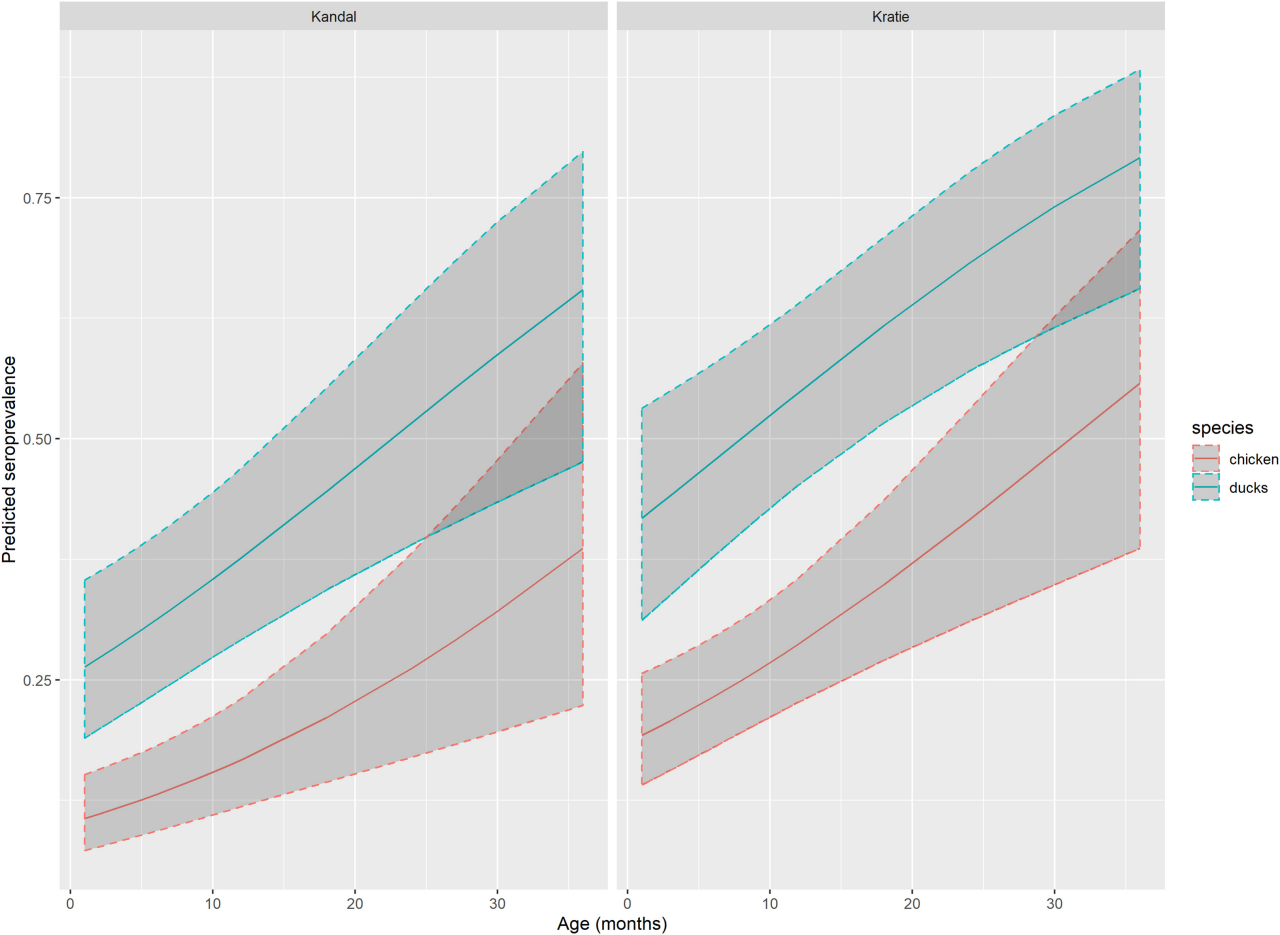
Flavivirus seroprevalence predicted by GLM. Predicted flavivirus seroprevalence in Kandal and Kratie provinces, for chicken (red line) and ducks (blue line) by age with 95% confidence interval (dark gray area) based on the generalized linear model.

### JEV- and WNV-Specific Seroprevalence Based on FRNT

**Figure 3:**
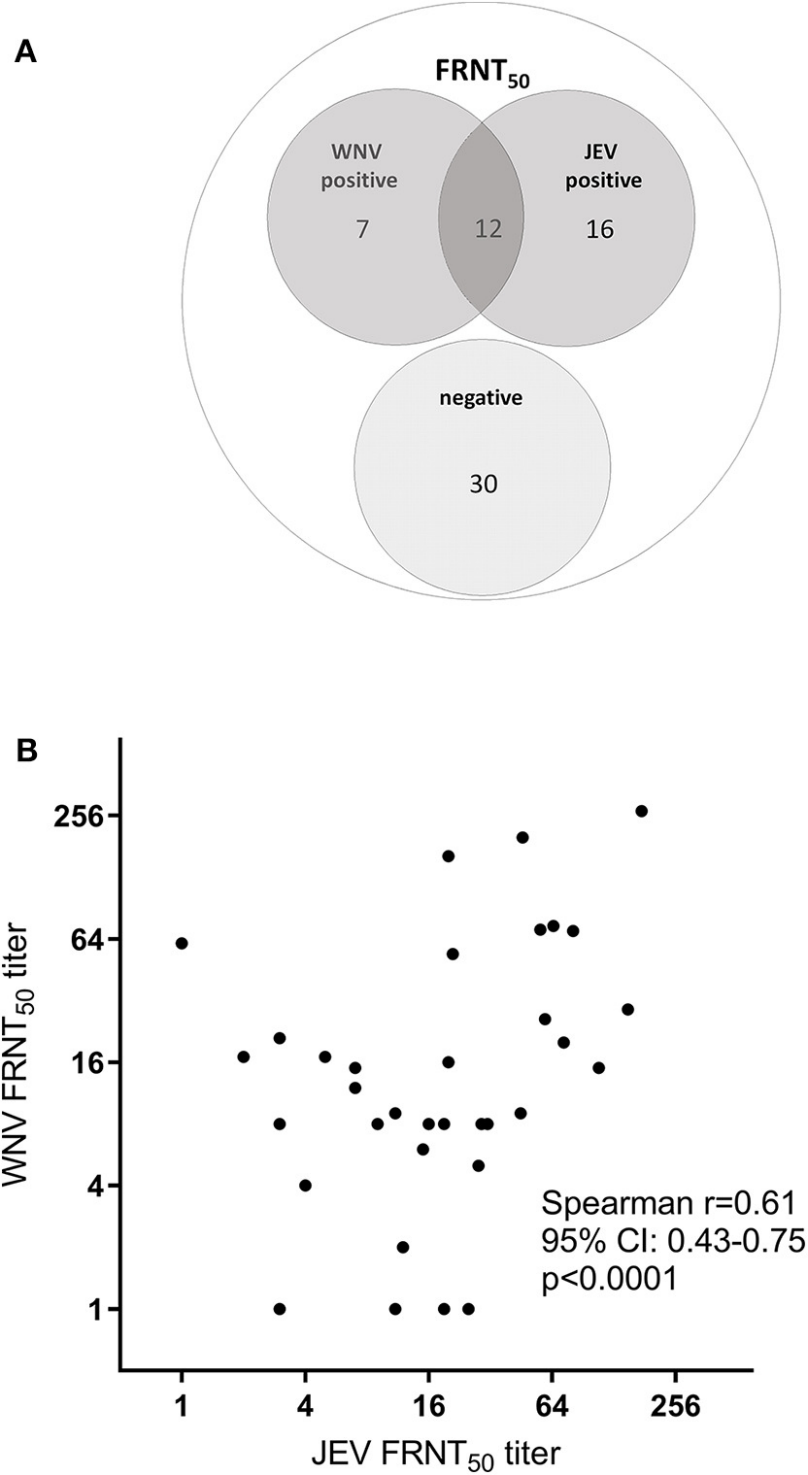
FRNT_50_ for JEV and WNV. A subset of 65 HIA-positive samples were analyzed for neutralizing antibodies against JEV and WNV by FRNT_50_. **(A)** Frequency of FRNT_50_ results. **(B)** Correlation of individual FRNT_50_ titer for JEV and WNV. Spearman correlation: *r* = 0.6397, *p* < 0.0001.

Among all the HIA-positive samples (*n* = 180), we analyzed a subset of 65 sera by foci reduction neutralization tests (FRNTs) against JEV and WNV based on criteria previously exposed (see Materials and Methods). The comparison of FRNT and HIA results showed that from the 65 samples tested positive for flavivirus hemagglutinating antibodies, only 35 had detectable levels of nAb for JEV and/or WNV (Table 4). From this subset, 28 samples showed nAb against JEV, including 12 sera that additionally had detectable levels of WNV nAb (Figure 3A). Interestingly, seven bird samples had nAb only against WNV and not against JEV. Most samples with detected nAb also showed HIA antibodies against more than one of the tested viruses (Table 4). Only one adult duck from Kandal with nAb against WNV was HIA positive exclusively against ZIKV.

**Table 4:** Comparison of HIA and FRNT results.

		**1**	**2**	**3**	**4**	**6**	**7**
	Number of positive results^*^	**Flavivirus HIA**	**JEV HIA**	**DENV ^**#**^ HIA**	**ZIKV HIA**	**JEV FRNT_**50**_^§^**	**WNV FRNT_**50**_^§^**
1	Flavivirus HIA	180	138	157	63	28	19
2	JEV HIA	138		118	60	21	18
3	DENV HIA^#^	157	118		53	26	15
4	ZIKV HIA	63	60	53		8	5
5	JEV & WNV FRNT_50_	35	27	31	10	28	19
6	JEV ${\text{FRNT}}_{50}^{§}$	28	21	26	8	-	12
7	WNV ${\text{FRNT}}_{50}^{§}$	19	18	15	5	12	-

§ *FRNT tested subset of 65 sera*.

* *HIA titer ≥80, FRNT_50_ titer ≥10*.

# *HIA for DENV-2 and/or DENV-3*.

The mean FRNT_50_ titer of JEV nAb (21.14; SD 35.08; 95% CI 12.45–229.83) was similar to the mean WNV FRNT_50_ titer (19.43; SD 46.89; 95% CI 7.81–31.05). The number of FRNT positive birds (Supplementary Table 2) and the levels of nAb (Supplementary Figure 3) did not significantly differ between poultry species or province of origin. Also, the nAb titers were significantly higher in the birds that were tested double positive for both JEV and WNV nAb compared to single positive sera (JEV *p* = 0.002; WNV *p* = 0.014; Mann-Whitney, Supplementary Figure 3C). We observed a weak correlation of the nAb titers between both viruses (Figure 3B; *r* = 0.64; *p* < 0.0001; Pearson correlation).

## Discussion

Our study found an overall flavivirus seroprevalence of 29% in domestic birds. This high percentage of seropositive poultry is highly likely due to the fact that JEV is endemic in Cambodia (31). This is similar to the findings of other JEV seroprevalence studies in Southeast Asia. In Bali (Indonesia) 20.6% of ducks and 36.7% of chickens were tested positive (37), and a study in Malaysia found 28.9% of the tested domestic birds positive for JEV antibodies (38). In addition, several experimental studies showed that domestic birds can be infected with JEV (5, 14, 15) and might even act as JEV reservoirs (39, 40). However, it is controversially discussed if they develop a sufficient viremia to infect mosquitoes (14, 41–43). In our study, ducks were more likely to be seropositive when they are 10 months or older than chickens (87.1% of ducks seropositive compared to 33.3% of chickens of that age). This could be due to feeding behavior of certain mosquitoes, different exposure due to distinct housing conditions or simply because ducks are usually kept longer before slaughtering than chickens.

Flavivirus detection in animals and humans especially in prevalence studies is mainly done serologically, as the viremic phase is rather short (44), e.g., JEV viremia lasts <1 week in chicks and ducklings (14). Yet, the co-circulation of several flaviviruses poses a diagnostic challenge due to the broad antibody cross-reactivity within and across the different serocomplexes (45). Indeed, extensive cross-reactivity is known for JEV and WNV even leading to reports of cross-protection (46, 47). Despite intensive attempts to develop specific diagnostic assays, the neutralization test is still considered to be the gold standard for the serological differentiation of flaviviruses (3). Due to the cross-reactivity, retrospective seroprevalence studies for flaviviruses are challenging in regions where more than one of these viruses circulate. The HIA is characterized by a high cross-reactivity which generally only allows a qualitative conclusion about the presence of flavivirus antibodies (48–51). Our HIA analysis and the moderate correlation of HIA titers among all viruses also demonstrated a high cross-reactivity especially between the two DENV serotypes 2 and 3 (Spearman *r* = 0.8999). In contrast, the correlation between the DENV HIA titers and the other viruses was less pronounced. This could be a consequence of the degree of antigenic similarities between the viruses (52), as the closely related DENV serotypes showed a high degree of correlation whereas JEV belongs to a different serocomplex than DENV and ZIKV. Additionally, DENV infection is not reported in poultry and therefore the antibodies measured against DENV might be the result of a non-specific immune response after a JEV infection. As a consequence of the endemicity of several flaviviruses in Cambodia, we chose a high threshold of ≥80 for HIA positivity. For the much more specific neutralization assay (FRNT), we chose the less stringent criteria for positivity by using the FRNT_50_ titer instead of FRNT_90_ and a threshold of ≥10 for positivity. This strategy was also used in other flavivirus seroprevalence studies in birds (48, 53, 54).

A limitation of our study is the uneven sample distribution regarding species, age and province of the animals. There were much less birds sampled in Mondulkiri province and ducks were overall underrepresented in the study cohort. We also had proportionally more samples from young chickens (1–3 months old) and older ducks (≥10 months; see Supplementary Table 1). Furthermore, we did not include WNV in the HIA because WNV is not endemic in Cambodia and therefore this virus is not part of our routine serological testing. Moreover, not all HIA positive samples could be tested with FRNT because of insufficient sera volume. Additionally, this assay is time- and labor-consuming. However, the samples analyzed with FRNT were not significantly different from the samples not tested with FRNT and from all samples that were tested positive for any flavivirus HIA (Supplementary Table 2), even if the HIA titers are slightly lower for the subset of FRNT samples compared to all HIA positive samples.

To our knowledge, this is the first serological evidence of WNV circulation in Cambodia, where the virus was last found before the 1980s (4). However, with our study we were only able to trace nAb against WNV in 7 domestic birds in the absence of JEV nAb. The direct detection of WNV in poultry, humans or mosquitoes as thorough evidence is still missing. The global distribution of WNV in tropical and temperate regions of Europe, Africa, the Americas, Western and central Asia is well-documented (17, 18, 55). In Southeast Asia, the main encephalitic flavivirus is still JEV (56). However, concerns about the ability of WNV to spread along bird migration routes are appropriate based on the recent expansion of WNV circulation in Eurasia (57) and the explosive dissemination of WNV in the Americas since the New York city outbreak in 1999 (58, 59). Importantly, despite their high serological cross-reactivity and virological similarities, JEV and WNV show distinct ecological and epidemiological specificities. Despite that both can be transmitted by *Culex* mosquitoes, the main vector of JEV is *Culex tritaeniorhynchus*, even as it was found in over 30 other mosquito species (9), whereas WNV is mainly transmitted by females of the *Culex pipiens* complex and their hybrids. For the endemic circulation of JEV, pigs play an important role as amplification hosts (60). In contrast, WNV can exclusively replicate in birds, especially in Passerines (61, 62).

Overall, recent studies investigated intensely the role of pigs in the JEV epidemiology as these are well-known amplification hosts for the virus. However, the contribution of poultry to the circulation of JEV remains understudied. Our study provides confirmation of a high seroprevalence for JEV in poultry as well as the first evidence of the circulation of WNV in domestic birds in the region. These findings may have consequences for the definition of areas at risk for JEV transmission, as the JEV might be able to circulate in areas with low densities of pigs or no pigs. This emphasizes the need for further and intensified surveillance of mosquito-transmitted diseases where backyard animals serve as potential amplification hosts.

## Data Availability Statement

The datasets generated for this study are available on request to the corresponding author.

## Ethics Statement

Ethical review and approval was not required for the animal study because there is no national (Cambodian) animal ethics committee. We followed the World Animal Health Organization (OIE) guiding principles on animal welfare included in the OIE Terrestrial Animal Health Code. All sampling campaigns were implemented with the supervision of the National Animal Health and Production Research Institute (NAHPRI), and local veterinary services. We provided the respective letter of approval from NAHPRI to the editorial office. Written informed consent was obtained from the owners for the participation of their animals in this study.

## Author Contributions

PD, JC, and VC: conceived and designed the study, the laboratory investigations and wrote the paper (comment and editing). A-SR and VC: sample collection and epidemiological analysis. HA, SI, and SM: performed the laboratory investigations. HA, A-SR, VD, and VC: analyzed the data. SS, ST, SI, VD, PD, and VC: contributed field work, reagents, materials, and analysis tools. HA, A-SR, and HL: wrote the paper (original draft).

### Conflict of Interest

The authors declare that the research was conducted in the absence of any commercial or financial relationships that could be construed as a potential conflict of interest.
