# Supplementary material for: Serological Evidence for Japanese Encephalitis and West Nile Virus Infections in Domestic Birds in Cambodia
Source: Front Vet Sci. 2020 Jan 29;7:15. doi: 10.3389/fvets.2020.00015 (PMC7000427; doi:10.3389/fvets.2020.00015)
Supplement: Supplementary file 1 [file Data_Sheet_1.docx]

Supplementary Material

# Supplementary Tables

## Supplementary Table 1: Age group distribution

|  | Chicken (n=417) | Ducks (n=203) | Total (n=620) |
| --- | --- | --- | --- |
| 1-3 month | 168 (40.3%) | 27 (13.3%) | 195 (31.5%) |
| 4-6 months | 92 (22.1%) | 52 (25.6%) | 144 (23.2%) |
| 7-9 months | 19 (4.6%) | 9 (4.4%) | 28 (4.5%) |
| ≥10 months | 67 (16.1%) | 80 (39.4%) | 147 (23.7%) |
| Unknown | 71 (17.0%) | 35 (17.2%) | 106 (17.1%) |

## Supplementary Table 2: Sample subsets

|  | FRNT samples  (HIA positive) | | HIA positive,  not-tested in FRNT | | all HIA positive | |
| --- | --- | --- | --- | --- | --- | --- |
| Total | 65 |  | 115 |  | 180 |  |
| Province |  |  |  |  |  |  |
| Kandal | 18 | 27.7% | 40 | 34.8% | 58 | 32.2% |
| Kratie | 27 | 41.5% | 71 | 61.7% | 98 | 54.4% |
| Mondulkiri | 20 | 30.8% | 4 | 3.5% | 24 | 13.3% |
| Species |  |  |  |  |  |  |
| Chicken | 39 | 60.0% | 60 | 52.2% | 99 | 55.0% |
| Duck | 26 | 40.0% | 55 | 47.8% | 81 | 45.0% |
| Age |  |  |  |  |  |  |
| 1-3 months | 12 | 18.5% | 21 | 18.3% | 33 | 18.3% |
| 4-6 months | 14 | 21.5% | 15 | 13.0% | 29 | 16.1% |
| 7-9 months | 0 | 0.0% | 6 | 5.2% | 6 | 3.3% |
| ≥10 months | 18 | 27.7% | 57 | 49.6% | 75 | 41.7% |
| unknown age | 21 | 32.3% | 16 | 13.9% | 37 | 20.6% |
| Mean HIA titer (95% CI) |  |  |  |  |  |  |
| JEV | 194.9 | (126.2 – 263.7) | 303.8 | (183.6 – 424.1) | 264.5 | (183.9 – 345.1) |
| DENV-2 | 395.4 | (156.7 – 634.1) | 708 | (394.8 – 1021) | 595.1 | (377.7 – 812.5) |
| DENV-3 | 568.6 | (124.2 – 1013) | 1059.4 | (496.6 – 1622) | 882.2 | (489.8 – 1275) |
| ZIKV | 105.1 | (53.54 – 156.6) | 146.3 | (76 – 216.5) | 131.4 | (83.1 – 179.7) |

## Supplementary Table 3: Japanese encephalitis and West Nile seroprevalence based on foci reduction neutralization test results (n=65)

| FRNT50 | negative | JEV positive | WNV positive | JEV + WNV positive | Total |
| --- | --- | --- | --- | --- | --- |
| Total number | 30 (46.2%) | 16 (24.6%) | 7 (10.8%) | 12 (18.5%) | **65** |
| Species |  |  |  |  |  |
| Chicken | 18 (46.2%) | 12 (30.8%) | 4 (10.3%) | 5 (12.8%) | **39** |
| Ducks | 12 (46.2%) | 4 (15.4%) | 3 (11.5%) | 7 (26.9%) | **26** |
| Age groups |  |  |  |  |  |
| 1-3 months | 6 (50.0%) | 1 (8.3%) | 2 (16.7%) | 3 (25.0%) | **12** |
| 4-6 months | 7 (50.0%) | 5 (35.7%) | 1 (7.1%) | 1 (7.1%) | **14** |
| 7-9 months | 0 - | 0 - | 0 - | 0 - | **0** |
| ≥10 months | 4 (22.2%) | 3 (16.7%) | 2 (11.1%) | 7 (38.9%) | **18** |
| Unknown | 11 (52.4%) | 7 (33.3%) | 2 (9.5%) | 1 (4.8%) | **21** |

# Supplementary Figure

## Supplementary Figure 1: Hemagglutination inhibition assay titers


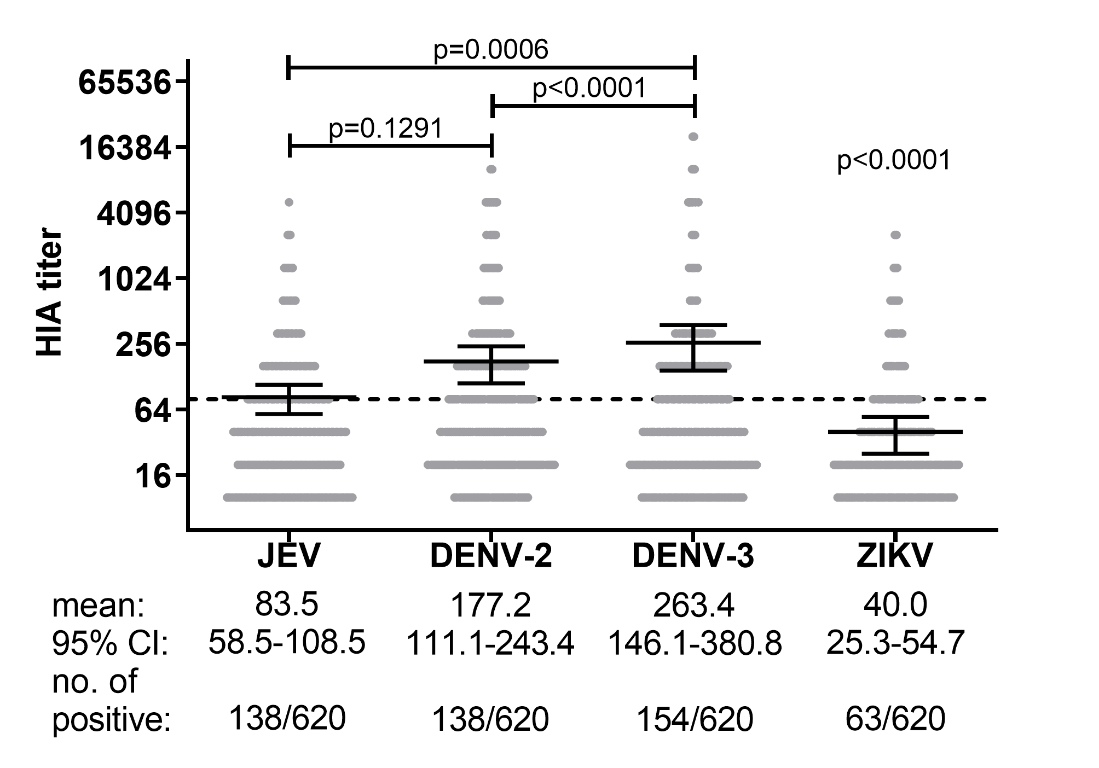


Individual HIA titers against Japanese encephalitis virus (JEV), dengue virus serotype 2 (DENV-2), dengue virus serotype 3 (DENV-3), and Zika virus (ZIKV) for all tested samples (n=620) with mean values and 95% CI. Due to the scaling of the y axis only values ≥10 are visualized in the graph. Dotted line shows threshold of 80 for positive HIA result. Analysis for significance was performed by Friedman test with Dunn’s multiple comparison test with GraphPad Prism 7.00.

## Supplementary Figure 2: Correlation plots of hemagglutination inhibition assay titers


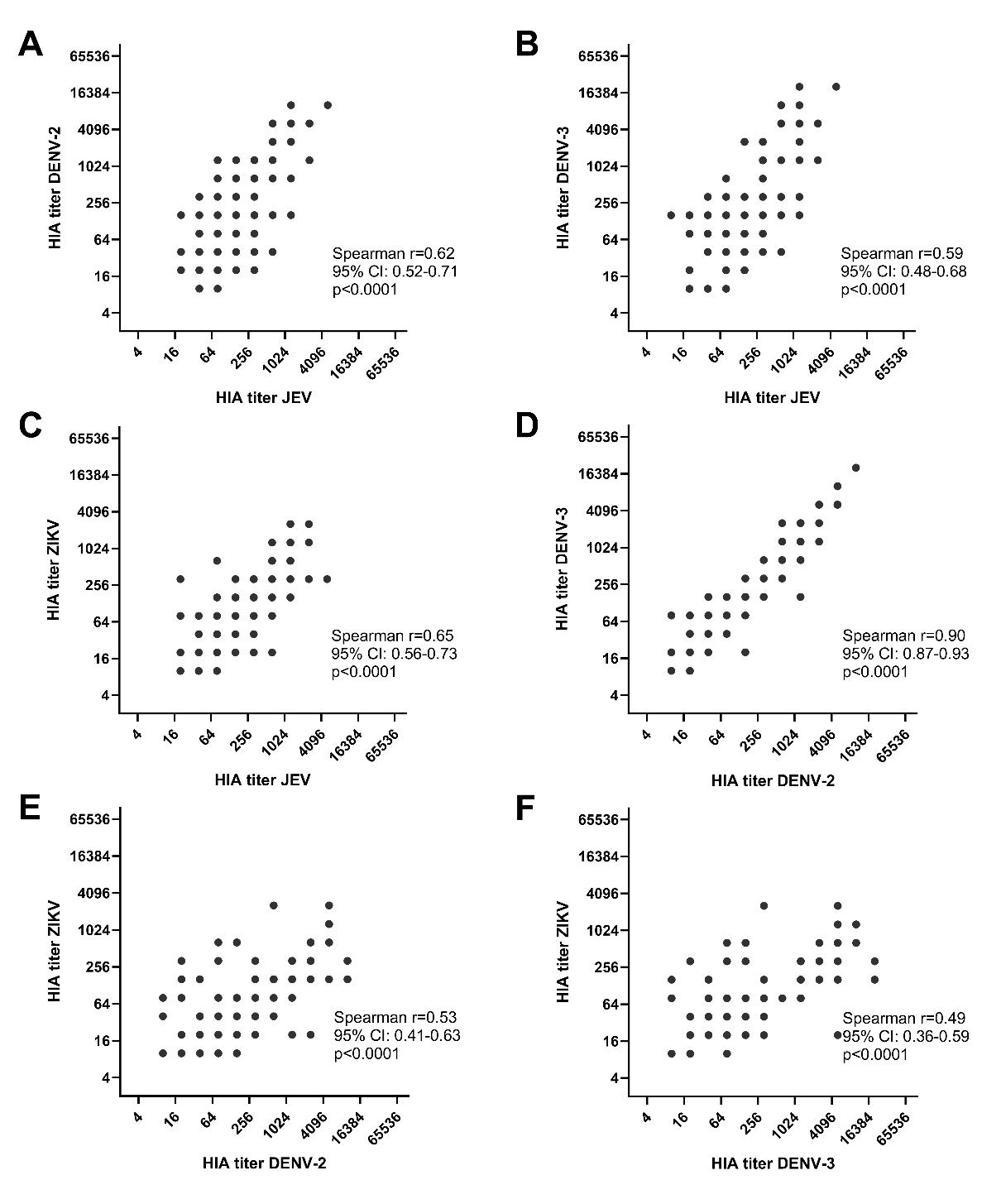


Correlation of individual HIA titers of all HIA-positive samples (n=180). (A) JEV titers vs DENV-2 titers, (B) JEV vs DENV-3, (C) JEV vs ZIKV, (D) DENV-2 vs DENV-3, (E) DENV-2 vs ZIKV, (F) DENV-3 vs ZIKV. Analysis for significance was performed by Spearman rank correlation with GraphPad Prism 7.00

## Supplementary Figure 3: FRNT_50_ analysis


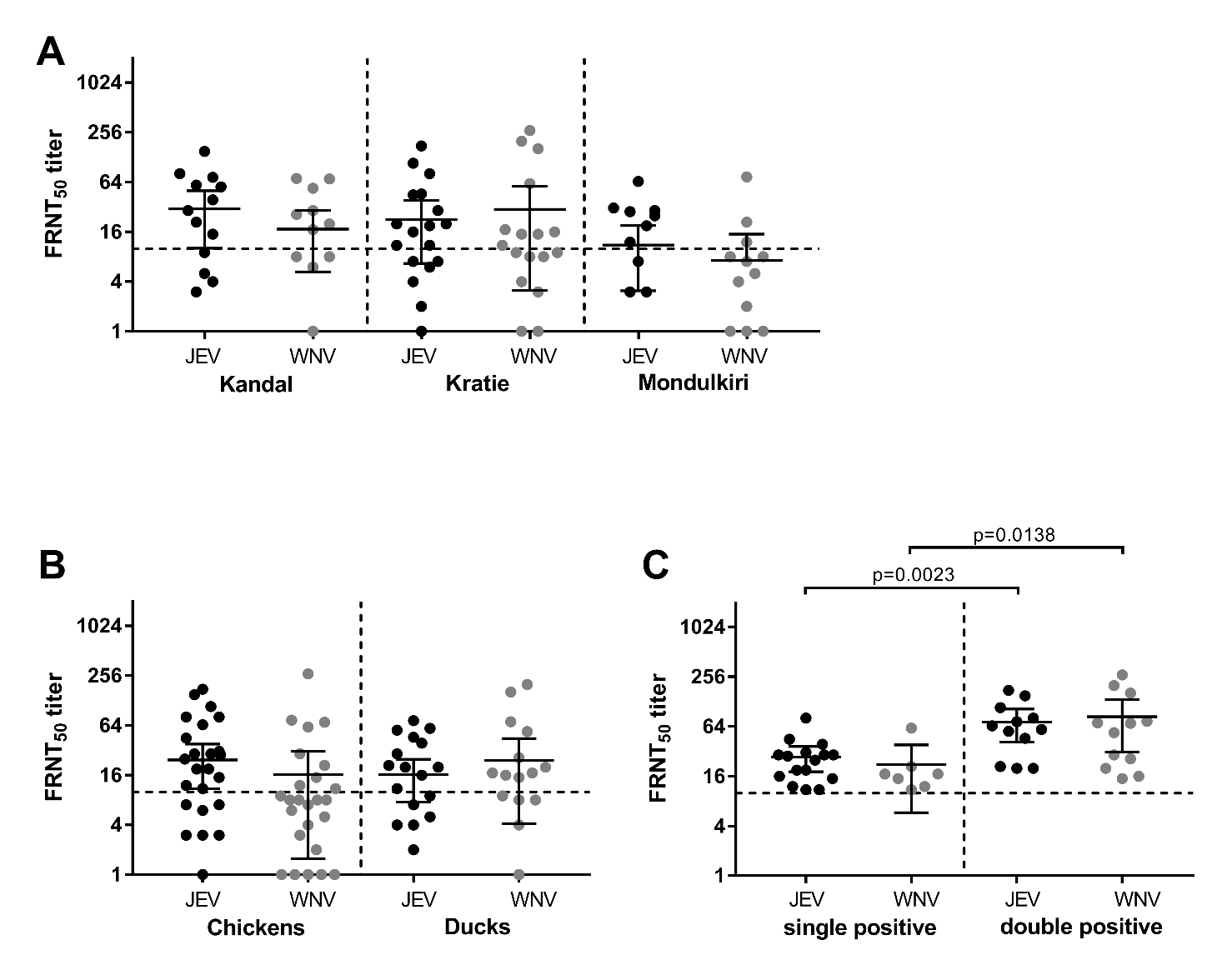


Individual FRNT_50_ titers against JEV (black) and WNV (grey) with mean and 95% confidence interval, differentiated by (**A**) province of origin of the domestic birds; (**B**) chicken and duck samples; (**C**) single JEV, single WNV and double JEV+WNV positive results . The threshold for positivity was FRNT_50_ ≥10 and is indicated by the dashed lines. Analysis for significance was performed by Mann-Whitney testing with GraphPad Prism 7.00.
